# Machine learning used to study risk factors for chronic diseases: A scoping review

**DOI:** 10.17269/s41997-025-01059-9

**Published:** 2025-06-11

**Authors:** Mahek Shergill, Steve Durant, Sharon Birdi, Roxana Rabet, Carolyn Ziegler, Shehzad Ali, David Buckeridge, Marzyeh Ghassemi, Jennifer Gibson, Ava John-Baptiste, Jillian Macklin, Melissa McCradden, Kwame McKenzie, Parisa Naraei, Akwasi Owusu-Bempah, Laura C. Rosella, James Shaw, Ross Upshur, Sharmistha Mishra, Andrew D. Pinto

**Affiliations:** 1 Upstream Lab, MAP Centre for Urban Health Solutions, Li Ka Shing Knowledge Institute, St. Michael’s Hospital, Toronto, ON Canada; 2 Michael G. DeGroote School of Medicine, McMaster University, Hamilton, ON Canada; 3 Health Science Library, Unity Health Toronto, Toronto, ON Canada; 4 Department of Epidemiology and Biostatistics, Western Centre for Public Health & Family Medicine, Western University, London, ON Canada; 5 Department of Health Sciences, University of York, York, UK; 6 WHO Collaborating Centre for Knowledge Translation and Health Technology Assessment in Health Equity, Ottawa, ON Canada; 7 Department of Epidemiology, Biostatistics and Occupational Health, School of Population and Global Health, McGill University, Montreal, QC Canada; 8 Department of Electrical Engineering and Computer Science (EECS), Institute for Medical Engineering & Science (IMES), MIT, Cambridge, MA USA; 9 Joint Centre for Bioethics, University of Toronto, Toronto, ON Canada; 10 Departments of Epidemiology & Biostatistics, Anesthesia & Perioperative Medicine, Schulich Interfaculty Program in Public Health, Western University, London, ON Canada; 11 Undergraduate Medical Education, Faculty of Medicine, University of Toronto, Toronto, ON Canada; 12 Department of Bioethics, The Hospital for Sick Children, Toronto, ON Canada; 13 Genetics & Genome Biology, SickKids Research Institute, Toronto, ON Canada; 14 Division of Clinical Public Health, Dalla Lana School of Public Health, University of Toronto, Toronto, ON Canada; 15 Wellesley Institute, Toronto, ON Canada; 16 CAMH, Toronto, ON Canada; 17 Department of Computer Science, Toronto Metropolitan University, Toronto, ON Canada; 18 Department of Sociology, Faculty of Arts & Sciences, University of Toronto, Toronto, ON Canada; 19 Institute for Better Health, Trillium Health Partners, Toronto, ON Canada; 20 Department of Physical Therapy, Faculty of Medicine, University of Toronto, Toronto, ON Canada; 21 Department of Family and Community Medicine, Faculty of Medicine, University of Toronto, Toronto, ON Canada; 22 Division of Infectious Diseases, Department of Medicine, Faculty of Medicine, University of Toronto, Toronto, ON Canada; 23 MAP Centre for Urban Health Solutions, Li Ka Shing Knowledge Institute, Unity Health Toronto, Toronto, ON Canada; 24 Institute of Medical Science, Faculty of Medicine, University of Toronto, Toronto, ON Canada; 25 Institute of Health Policy, Management and Evaluation, and Division of Epidemiology, Dalla Lana School of Public Health, University of Toronto, Toronto, ON Canada; 26 ICES, Toronto, ON Canada; 27 Department of Family and Community Medicine, St. Michael’s Hospital, Toronto, ON Canada

**Keywords:** Machine learning, Artificial intelligence, Public health, Population health, Risk factors, Non-communicable diseases, Apprentissage automatique, Intelligence artificielle, Santé publique, Santé de la population, Facteurs de risque, Maladies non transmissibles

## Abstract

**Objectives:**

Machine learning (ML) has received significant attention for its potential to process and learn from vast amounts of data. Our aim was to perform a scoping review to identify studies that used ML to study risk factors for chronic diseases at a population level, notably those that incorporated methods to mitigate algorithmic bias. We focused on ML applications for the most common risk factors for chronic disease: tobacco use, alcohol use, unhealthy eating, physical activity, and psychological stress.

**Methods:**

We searched the peer-reviewed, indexed literature using Medline (Ovid), Embase (Ovid), Cochrane Central Register of Controlled Trials and Cochrane Database of Systematic Reviews (Ovid), Scopus, ACM Digital Library, INSPEC, and Web of Science’s Science Citation Index, Social Sciences Citation Index, and Emerging Sources Citation Index. Among the included studies, we examined whether bias was considered and identified strategies employed to mitigate bias.

**Synthesis:**

The search identified 10,329 studies, and 20 met our inclusion criteria. The studies we identified used ML for a wide range of goals, from prediction of chronic disease development to automating the classification of data to identifying new associations between risk factors and disease. Nine studies (45%) included some discussion of algorithmic bias. Studies that incorporated a broad array of sociodemographic variables did so primarily to improve the performance of a ML model rather than to mitigate potential harms to populations made vulnerable by social and economic policies.

**Conclusion:**

This work contributes to our understanding of how ML can be used to advance population and public health.

**Supplementary Information:**

The online version contains supplementary material available at 10.17269/s41997-025-01059-9.

## Résumé

**Objectifs:**

L’apprentissage automatique (AA), ou “machine learning” (ML), est largement étudié en raison de sa capacité à traiter et à apprendre à partir de grandes quantités de données. Notre objectif était de réaliser une revue de la portée pour identifier les recherches qui ont utilisé l’AA pour explorer les facteurs de risque des maladies chroniques au niveau de la population. Nous nous sommes concentrés sur les applications de l’AA aux facteurs de risque les plus courants: la consommation de tabac, la consommation d’alcool, une alimentation malsaine, le manque d’activité physique et le stress psychologique.

**Méthodes:**

Nous avons effectué une recherche dans des bases de données scientifiques reconnues telles que Medline, Embase, le Registre central Cochrane, Scopus, ACM Digital Library, INSPEC, et divers indices du Web of Science. Pour les études identifiées, nous avons examiné si elles considéraient les biais et quelles stratégies elles utilisaient pour les atténuer.

**Synthèse:**

Nous avons identifié 10,329 études, dont 20 répondaient à nos critères d’inclusion. Ces études ont utilisé l’AA à divers fins: prédire le développement de maladies chroniques, automatiser la classification des données, ou identifier de nouvelles associations entre facteurs de risque et maladies. Neuf études (45%) ont traité la question des biais. Celles qui incluaient un large éventail de variables sociodémographiques l’ont principalement fait pour améliorer les performances des modèles d’AA, plutôt que pour réduire les dommages potentiels pour les populations vulnérables résultant des politiques sociales et économiques.

**Conclusion:**

Cette étude contribue à une meilleure compréhension de l’utilisation potentielle de l’apprentissage automatique pour améliorer la santé publique.

## Introduction

Non-communicable diseases (NCDs), such as cancer, diabetes, and cardiovascular and chronic respiratory diseases, are the leading cause of death worldwide, with a disproportionately higher burden of mortality in low- and middle-income countries (LMICs) (Murray, 2024). The key risk factors for these NCDs include tobacco use, unhealthy eating, alcohol consumption, physical inactivity, and psychological stress (Glasgow & Schrecker, 2015). Managing risk factors is crucial for the prevention of NCDs and is a key area for public health action (Squires et al., 2023).

Artificial intelligence (AI) refers to the simulation of human intelligence by machines and computer systems (Davenport & Kalakota, 2019). It encompasses various technologies designed to perform tasks typically requiring human cognition, such as learning, reasoning, problem-solving, decision-making, and natural language understanding (Norori et al., 2021). Machine learning (ML) is a subset of AI that uses statistical techniques to enable computer systems to learn patterns and make predictions or decisions without being explicitly programmed (Helm et al., 2020). By analyzing and training on large datasets, ML models can improve their performance over time (Purushotham et al., 2018). In healthcare, ML is commonly applied in precision medicine, while it predicts effective treatment protocols based on patient characteristics and treatment contexts. ML is increasingly being used in population and public health to analyze large datasets, identify health trends, predict disease outbreaks, and inform policy decisions for improving community health outcomes (Morgenstern et al., 2020).

However, ML can perpetuate algorithmic biases that exacerbate health inequities along socioeconomic, racial, ethnic, and gender lines (Buckeridge, 2020; Chen et al., 2019; Practical guidance on artificial intelligence for health-care data, (2019); Topol, 2019). In supervised learning, which involves training an algorithm on labelled datasets to classify data or predict outcomes, biases can enter the model through incomplete training data (i.e., non-random instances of absent points), leading to inaccurate predictions for diverse populations. In unsupervised learning, which detects underlying patterns using unlabelled data, biases can emerge from the data (i.e., insufficient representation of certain groups), reflecting underlying social biases against the studied population (Benjamin, 2019; Gianfrancesco et al., 2018; Rajkomar et al., 2018, 2019).

While ML has garnered attention for its applications in clinical care, its potential to address public health challenges, particularly for NCD prevention, has been insufficiently explored (Mhasawade et al., 2021). Previous reviews have primarily focused on disease prediction or on specific ML methodologies, such as the application of ensemble models for diabetes or high-dimensional feature selection in cancer research. Our study takes a broader, population-level perspective by concentrating on behavioural and psychosocial risk factors for chronic diseases—tobacco use, alcohol use, unhealthy eating, physical activity, and psychological stress (Ganie et al., 2023; Zanella et al., 2022). While prior work often emphasized technical advancements or the optimization of model performance metrics, our review uniquely integrates an analysis of the ethical considerations surrounding bias and the sociodemographic variables used (Alanazi, 2022; Mishra et al., 2020).

The definition of public health guides our focus in this review as “the science and art of preventing disease, prolonging life, and promoting physical and mental health through organized community efforts” (Winslow, 1920).This perspective emphasizes population-level interventions and strategies aimed at improving health outcomes across entire communities rather than focusing on individual-level or clinical care. While clinical applications are integral to overall health systems, they align more closely with direct patient care and treatment rather than the broader community-based efforts central to public health. By concentrating on these population-level approaches, we aim to provide insights that support systemic changes and large-scale prevention efforts consistent with the foundational principles of public health. To address this, it is essential to synthesize evidence on the use of ML in studying risk factors for NCDs. This includes examining how algorithmic biases, such as those related to socioeconomic or demographic inequities, are identified and addressed during the development of ML models.

To address the limited application of ML in public health, we conducted a scoping review to identify studies that utilize ML to examine risk factors for NCDs. Specifically, we focused on common health behaviours associated with chronic diseases, including tobacco use, unhealthy diets, physical inactivity, alcohol consumption, and psychological stress. In addition, we assessed whether and how algorithmic biases—such as those related to socioeconomic, racial, or gender inequities—manifest during the design, training, and implementation of ML models and evaluated how developers addressed these biases.

## Methods

Our protocol was published by the Open Science Framework on December 23, 2021 (available at https://osf.io/czyte/). Our reporting of this scoping review followed the Preferred Reporting Items for Systematic Reviews and Meta-Analyses Extension for Scoping Reviews (PRISMA-ScR) (Tricco et al., 2018).

### Search methodology

We searched Medline (Ovid), Embase (Ovid), Cochrane Central Register of Controlled Trials and Cochrane Database of Systematic Reviews (Ovid), Scopus, ACM Digital Library, INSPEC, and Web of Science’s Science Citation Index, Social Sciences Citation Index, and Emerging Sources Citation Index on December 23 and 24, 2021, for the period 1946 to 2021. Additionally, a hand search was performed in September of 2022.

An information specialist with Library Services, Unity Health Toronto (CZ) searched using a combination of subject headings and keywords, translated for each database, for the broad concepts of ML combined with risk factors for chronic disease, which include smoking/vaping, alcohol drinking, physical inactivity, unhealthy eating, or psychological stress. The Non-Communicable Disease Alliance identified the aforementioned four behavioural risks as having the greatest impact on the burden of disease on a global scale (NCD Alliance, 2017). Additionally, we are including psychological stress based on existing literature that recognizes it as a significant factor for NCDs, which can be mitigated via population- and public health interventions (Caruso et al., 2018; Harris et al., 2017; McEwen & Stellar, 1993; Steptoe & Kivimäki, 2012). The full search strategy is available in Supplementary File 1.

Duplicates were removed through DistillerSR, and all citations were then uploaded. Each citation was reviewed by two people independently (MS and PC). Reviewers had previous experience in the analysis of ML applications. Any citations where there was a difference of opinion were brought to the study team to discuss, and the principal investigator (ADP) decided on final inclusion or exclusion.

### Eligibility criteria

Studies were included that used ML models applied in population and public health, specifically regarding health behaviours (smoking/vaping, alcohol drinking, physical inactivity, unhealthy eating) and physiological stress. This included population-wide approaches (fully implemented or piloting for this purpose), even if delivered through clinical settings (e.g., population-wide screening), as well as subsets of the general population at specific points in the life course (i.e., seniors, children). We included studies that discussed ML models, specifically at the stages of model development, model training, and model implementation. Model development included defining the problem, identifying the data required, preparing the data, and assigning some data for training and some for validation (Rajkomar et al., 2019). Model training included running data from the training dataset through the model and comparing the model’s predicted label with a “ground-truth” label (supervised) and updating the model parameters to make subsequent predictions more correct or analyzing unlabelled data to identify groups that have similar features (unsupervised machine learning) (Rajkomar et al., 2019). Model implementation included using the model on a validation dataset using parameters such as accuracy, area under the receiver operator curve, sensitivity and specificity, and gain and lift (Rajkomar et al., 2019). From a contextual standpoint, we included articles with a global scope, without imposing country restrictions, focusing specifically on ML models that incorporate data and address algorithmic bias related to race, ethnicity, sex, gender, and socioeconomic status.

We excluded studies that focused on individual-level or clinical care applications of ML, as our focus was on population-level applications. Additionally, we excluded studies examining the etiology, progression, or complications of diseases, as these domains fall outside the scope of broader public health strategies. Domains not traditionally part of public health systems, such as occupational health, were also excluded to maintain our focus on general population-level health issues. We further excluded studies targeting high-risk groups or specialized medical settings (e.g., smokers or hospital patients), as well as those limited to subsets of the population defined by sociodemographic characteristics other than age (e.g., ethnicity, sex). High-risk groups and specialized settings often have unique health profiles and intervention responses that may not reflect the experiences of the general population, while studies focused on specific sociodemographic subsets may introduce biases that could obscure overall applicability. This approach helps ensure that our analysis remains widely representative and relevant to public health strategies and interventions. Finally, non-peer-reviewed materials, including commentaries, editorials, analyses, theses, and conference proceedings, were excluded to ensure the inclusion of high-quality, peer-reviewed evidence.

### Study selection and data collection process

The full text of each citation that met our criteria was reviewed again by a single member of the study team (MS) to ensure it fit our criteria and then proceeded to data extraction. One member of the study team assisted with data extraction (MS). We developed and used a data extraction table to collect and summarize data from each article (Table 1). We collected data on title, journal, year, ML application type, intended purpose, study design, research question(s), intervention, results, jurisdiction, data source, units of analysis, sample size, demographics, identification of any potential bias in the ML model, algorithmic (biases related to gender, sex, ethnicity, socioeconomic status, and LMIC transferability), potential bias mitigation strategies, risk factor studied, target population and setting, intended users, and impact. If information was not available from an article, its authors were contacted through email to clarify any points. To synthesize our findings, we performed a narrative synthesis. This scoping review aimed to identify and describe patterns in how biases are introduced and mitigated in ML studies (Popay et al., 2006). Given this focus on synthesizing descriptive patterns rather than critically appraising methodological rigour, as in a systematic review, a comprehensive quality assessment across all domains of ML studies was not deemed necessary (Mak & Thomas, 2022).

**Table 1:** Summary of included studies

Author(s)	Machine learning application	Year	Study design	Aim	Jurisdiction	Data source	Risk factor	Bias identification	Bias mitigation
Afzali	Multiple: LR, SVM, RF, NN, LASSOR, RR, EN	2018	Cross-sectional	To predict levels of alcohol use in adolescents	Canada and Australia	Co-venture cohort	Alcohol use	No	No
Al-Jebrni	DNN	2020	Experimental	Objective quantification of stress from selfie videos	110 countries	*Wildflowers Mindfulness* app selfie videos	Psychological stress	No	No
Ali	Gradient-boosted ML	2022	Cohort	Predict obesity and smoking	Australia	D Health Trial and QSkin Study	Smoking/vaping	No	No
Allahbakshi	RF	2021	Experimental	Using accelerometer data to detect activity levels	NR	MOASIS Study	Physical inactivity	No	No
Allem	Gephi visualization algorithm	2017	Thematic analysis	Identify priorities from e-cigarette discussion on Twitter	E-cigarette Twitter posts	Twitter	Smoking/vaping	No	No
Amialchuk	Extreme Gradient-boosted regression, RF	2021	Cross-sectional	ML to fit model of social interactions in alcohol consumption	USA	National Longitudinal Study of Adolescent to Adult Health	Alcohol use	No	No
Aschbacher	Ensemble tree algorithm	2021	Experimental	Predict individual’s breath alcohol concentrations	Predominantly USA but global	Breath alcohol data provided to the University of California, San Francisco	Alcohol use	No	No
Atuegwu	Boruta, LAS, LASSO	2020	Observational	Determine factors associated with e-cigarette use in youth	USA	2016 and 2017 behavioural risk factor surveillance system	Smoking/vaping	Yes: sex, ethnicity, SES	No
Atuegwu	LASSO	2021	Cross-sectional	Associations between different types of disability and e-cigarette use	USA	2016 and 2017 behavioural risk factor surveillance system	Smoking/vaping	Yes: sex, ethnicity, SES	No
Bae	NLP	2021	Experimental	Automatic classification of smoking status from unstructured EHR	Korea	Clinical notes from Seoul National University Hospital	Smoking/vaping	No	No
Bonnell	RF	2020	Cross-sectional	Clinical prediction tool for unhealthy drinking based on routine demographic and laboratory data	USA	1996–2016 National Health and Nutrition Examination Survey	Alcohol use	Yes: ethnicity	Yes: study states that though race and ethnicity are associated with alcohol use, they were removed a priori due to common misclassification problems, especially in EHR data
Caccamisi	SVM, and SMO	2020	Cross-sectional	Automatic classification of unstructured information on smoking status from EMR data	Sweden	Swedish EMR data	Smoking/vaping	No	No
Dadi	NR	2021	Cross-sectional	Build proxy measures of psychological constructs	UK	UK Biobank	Psychological stress	Yes: sex, ethnicity, SES	No
Fu	RF	2022	Cohort	Predict frequent vaping status and identify contributing factors, vulnerable populations	CA, USA	Happiness and Health Study Wave 7 Survey ever vaping 12th Grade participants	Smoking/vaping	Yes: sex, ethnicity, SES	No
Han	LR	2021	Cohort	Identifies emerging predictors of adolescent electronic nicotine delivery systems	USA	Wave 1–4 of youth public files for the Population Assessment of Tobacco and Health Study	Smoking/vaping	No	No
Júdice [37]	Decision tree technique	2021	Cross-sectional	Determine correlates which best predict sensor-based physical activity, sedentary time, and self-reported cell phone screen time	Portugal	Subset of nationwide survey examining physical activity levels using sensor-based data	Physical inactivity	Yes: sex, SES	Yes: socioecologic framework used to hierarchize the correlates that best predict screen time and physical activity, while also considering intrapersonal, interpersonal, and neighbourhood-physical environmental factors (safety, proximity to parks, violence)
Nakandala [35]	CNN	2021	Cross-sectional	Evaluation of a CNN model on a free living dataset and compare with ML algorithms	USA	Women enrolled in study of sedentary behaviour and breast cancer	Physical inactivity	No	No
Oku [40]	Gradient-boosted ML	2020	Cross-sectional	Combine ML methods and graph analysis to build predictive networks to the National Student Health Survey	Brazil	National Survey of Students’ Health	Unhealthy eating	Yes: sex, ethnicity, SES	No
Raghupathi [30]	Multilayer Perceptron feed-forward neural network	2017	Cross-sectional	Explore association between behavioural habits and chronic disease	USA	2012 behavioural risk factor surveillance system	Alcohol use, unhealthy eating, physical activity	Yes: sex, SES	No
Young Wolff [22]	NLP	2017	Cross-sectional	Examine documented electronic nicotine delivery systems in the EHR	CA, USA	Kaiser Permanente Northern California patients	Smoking/vaping	Yes: sex, ethnicity	No

*LR*, logistic regression; *SVM*, support vector machine; *RF*, random forest; *NN*, neural network; *LASSOR*, lasso regression; *RR*, ridge regression; *EN*, elastic-net; *DNN*, deep neural network; *MOASIS*, Mobility, Activity, and Social Interactions Study; *NR*, not reported; *LAS*, least absolute shrinkage; *LASSO*, least absolute shrinkage and selection operator; *SES*, socioeconomic status; *NLP*, natural language processing; *EHR*, electronic health record; *SMO*, sequential minimal optimization; *CNN*, convolutional neural network

### Algorithmic bias identification

In the context of this scoping review, algorithmic bias is defined according to Panch et al. as: “the instances when the application of an algorithm compounds existing inequities in socioeconomic status, race, ethnic background, religion, gender, disability or sexual orientation to amplify them and adversely impact disparities in health systems” (Panch et al., 2019). We assessed studies to identify biases related to gender, sex, ethnicity, socioeconomic status, and LMIC transferability. As specified by Panch et al., for ML models to achieve accuracy, fairness, and equity, it is imperative to account for contextual factors such as geopolitical, economic, and cultural influences (Panch et al., 2019). These factors play a critical role in shaping the datasets used for model training and directly influence the outcomes of ML systems (Mittermaier et al., 2023a). Neglecting these dimensions can result in models that perpetuate existing biases, reinforce inequities, and fail to address the needs of underrepresented populations (Howard & Borenstein, 2018). For example, health systems across the world vary significantly in their design, resources, and patient demographics, with diverse socioeconomic and cultural contexts influencing health outcomes (Panch et al., 2019). By integrating these contextual elements, AI models can better represent the populations they aim to serve, ensuring that predictions and decisions are both inclusive and relevant (Panch et al., 2019). This approach not only enhances the ethical and practical application of AI but also strengthens its potential to reduce disparities and promote equity in health systems and beyond (Howard & Borenstein, 2018).To ensure robust and equitable model development, it is essential to utilize datasets that include individuals from diverse socioeconomic backgrounds, cultural contexts, and genetic profiles (Panch et al., 2019). Consequently, biases related to gender, sex, ethnicity, and socioeconomic status were specifically analyzed to determine whether studies acknowledged the underrepresentation of certain groups in datasets and the disproportionate frequency with which they are sampled. Additionally, the transferability of findings to LMICs was evaluated to assess the broader applicability of the models.

## Results

**Fig. 1:**
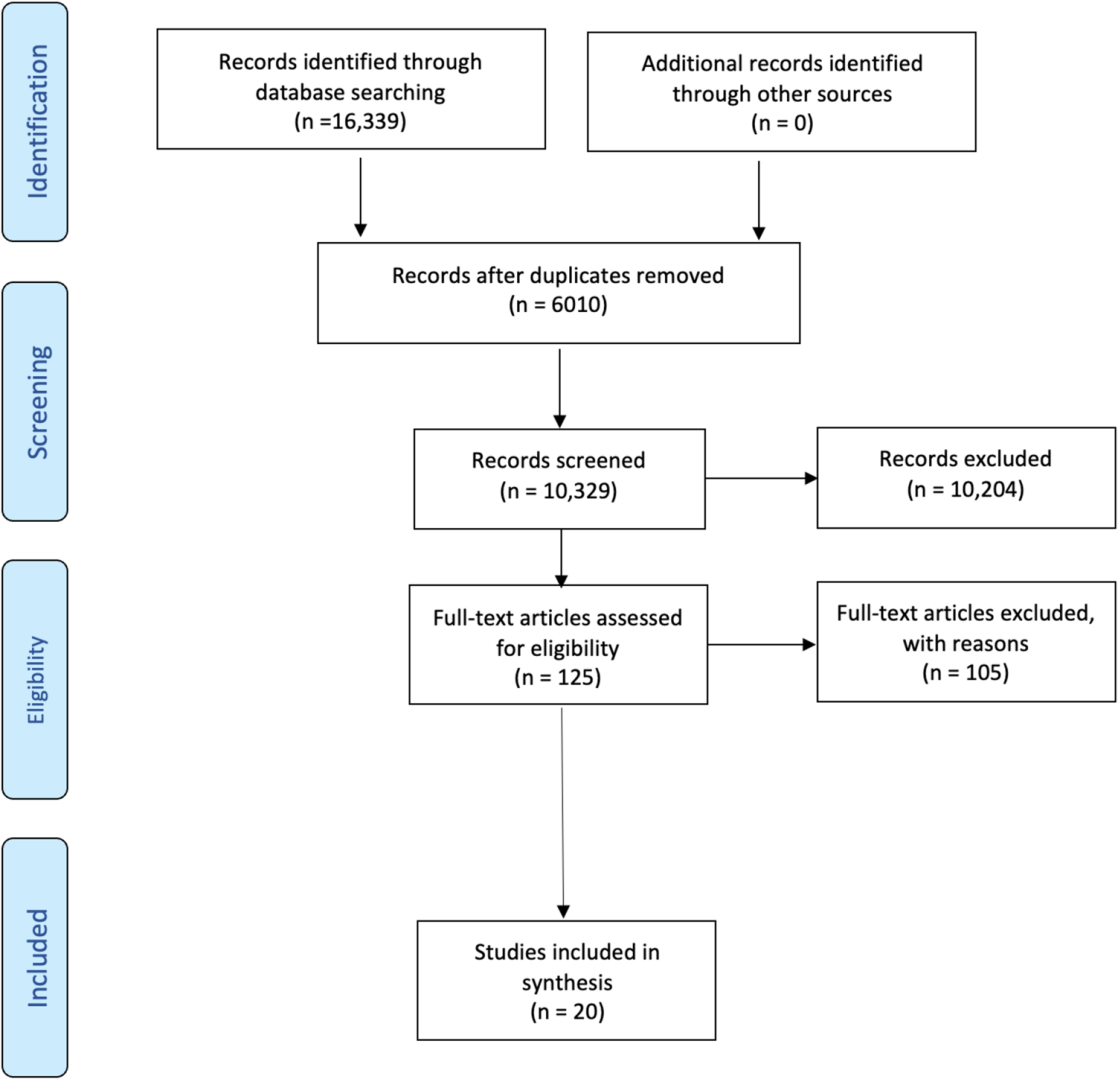
PRISMA flow diagram

In total, 10,329 citations were identified through the search (Fig. 1). After title and abstract screening, 125 articles were selected for full-text review, resulting in 20 articles being included in the final analysis. The articles explored various risk factors for NCDs, with the distribution of risk factors as follows: smoking/vaping (*n* = 9, 45%) (Ali et al., 2022; Allem et al., 2017; Atuegwu et al., 2020, 2021; Bae et al., 2021; Caccamisi et al., 2020; Han et al., 2021; Young-Wolff et al., 2017), alcohol use (*n* = 5, 25%) (Afzali et al., 2019; Amialchuk et al., 2021; Aschbacher et al., 2021; Bonnell et al., 2020; Raghupathi & Raghupathi, 2017), physical inactivity (*n* = 3, 15%) (Allahbakhshi et al., 2021; Júdice et al., 2021; Nakandala et al., 2021), psychological stress (*n* = 2, 10%) (Al-Jebrni et al., 2020; Dadi et al., 2021), and unhealthy eating (*n* = 1, 5%) (Oku et al., 2019). The included articles described associative models (16/20, 80%) and predictive models (4/20, 20%).

The primary reasons for exclusion included studies that referenced a risk factor of interest but did not focus explicitly on analyzing the factor itself. For instance, some studies assessed smoking or alcohol use within broader models addressing cardiovascular disease risk (Drożdż et al., 2022; Foldyna et al., 2024; Peng et al., 2023). Other articles were not conducted with a population-level focus and were developed for a specific clinical setting (Alowais et al., 2023). All research papers were published between 2017 and 2022. For characteristics of included studies, see Table 1.

### Countries represented

Source countries for the studies in this review included the United States (*n* = 10, 50%) (Allem et al., 2017; Amialchuk et al., 2021; Atuegwu et al., 2020, 2021; Bonnell et al., 2020; Fu et al., 2022; Han et al., 2021; Nakandala et al., 2021; Raghupathi & Raghupathi, 2017; Young-Wolff et al., 2017), Australia (*n* = 1, 5%) (Ali et al., 2022), the United Kingdom (*n* = 1, 5%) (Dadi et al., 2021), Korea (*n* = 1, 5%) (Bae et al., 2021), Sweden (*n* = 1, 5%) (Caccamisi et al., 2020), Portugal (*n* = 1, 5%) (Júdice et al., 2021), and Brazil (*n* = 1, 5%) (Oku et al., 2019). Three studies were conducted in multiple countries (*n* = 3, 15%) (Afzali et al., 2019; Al-Jebrni et al., 2020; Aschbacher et al., 2021) and one study’s location was unspecified (*n* = 1, 5%) (Allahbakhshi et al., 2021).

#### Machine learning applications

The studies employed a wide range of ML methods, including deep neural networks, gradient-boosted ML, random forest, Gephi visualization algorithm, ensemble tree algorithm, Boruta, least absolute shrinkage and selection operator, natural language processing, support vector machine sequential minimal optimization, logistic regression, decision tree, convolutional neural network, multilayer perceptron feed-forward neural network, and support vector machine. Included studies were categorized by intended purpose: surveillance (*n* = 2, 10%) (Al-Jebrni et al., 2020; Allem et al., 2017), predicting disease incidence in the population (*n* = 0), predicting risk in the population (*n* = 14, 70%) (Ali et al., 2022; Amialchuk et al., 2021; Aschbacher et al., 2021; Atuegwu et al., 2020, 2021; Bonnell et al., 2020; Caccamisi et al., 2020; Dadi et al., 2021; Fu et al., 2022; Han et al., 2021; Júdice et al., 2021; Oku et al., 2019; Raghupathi & Raghupathi, 2017; Young-Wolff et al., 2017), evaluating effectiveness of an intervention (*n* = 3, 15%) (Allahbakhshi et al., 2021; Bae et al., 2021; Nakandala et al., 2021), or a comparison of models/approaches (*n* = 1, 5%) (Afzali et al., 2019). For instance, Atuegwu et al. used ML models on data from the Behavioral Risk Factor Surveillance System, which included 79,539 participants, to identify factors associated with e-cigarette use, showcasing the potential of ML in large-scale risk prediction (Atuegwu et al., 2020, 2021).

### Data sources

The study populations demonstrated considerable variation. Five studies specifically targeted adolescents, leveraging data collected through school-based surveys. Among these, the Canadian Co-Venture cohort examined the effectiveness of drug and alcohol prevention programs in adolescents (Afzali et al., 2019; Amialchuk et al., 2021; Fu et al., 2022; Han et al., 2021; Oku et al., 2019). Additionally, national study cohorts were prominently represented, with data sourced from the UK Biobank, the National Longitudinal Study of Adolescent to Adult Health, the Behavioral Risk Factor Surveillance System, and the National Health and Nutrition Examination Survey (Amialchuk et al., 2021; Atuegwu et al., 2020, 2021; Bonnell et al., 2020; Dadi et al., 2021). One study utilized electronic health records from the Kaiser Permanente medical system to evaluate relevant outcomes (Young-Wolff et al., 2017).

#### Strategies used to identify and mitigate algorithmic bias

Algorithmic bias was addressed in nine (45%) of the included studies. However, these discussions were generally superficial and focused on recall and misclassification bias, often failing to consider algorithmic bias as a rationale for specific model training, development, or implementation decisions. The remaining studies focused on mitigating traditional biases, such as misclassification, recall bias, and non-response bias (Young-Wolff et al., 2017).

For instance, Afzali et al. (2019) reduced recall bias by utilizing longitudinal data from the Canadian Co-Venture cohort. This approach involved repeated measures of alcohol use among adolescents, thereby minimizing reliance on retrospective self-reporting. Similarly, Han et al. (2021) addressed misclassification bias through a rigorous data validation process that ensured e-cigarette use categories were accurately defined and consistently applied across their dataset. Nakandala et al. (2021) mitigated non-response bias by employing data imputation techniques, allowing for the inclusion of incomplete data from physical activity datasets to maintain representativeness.

Some studies adopted innovative strategies that indirectly minimized potential bias by examining how health behaviours are influenced by interpersonal factors and the built environment. For example, Fu et al. emphasized the importance of recognizing intersectionality—the interplay of multiple sociodemographic factors—in influencing the risk of frequent vaping. The study incorporated nine variables, including age, gender, race/ethnicity, neighbourhood characteristics (e.g., median household income, high school graduation rates, unemployment rates), school-related variables (e.g., percentage of free or reduced-cost lunch recipients), and perceived discrimination. Fu et al. found a strong relationship between perceived discrimination and frequent vaping, with the likelihood of vaping increasing alongside experiences of discrimination across all ages. They also identified a particularly high-risk subgroup: students younger than their classmates who reported moderate to high levels of discrimination. This study highlights the value of including and pairing sociodemographic variables in ML models to identify vulnerable subgroups and tailor interventions effectively.

Additionally, Júdice et al. applied a socioecological model to assess physical inactivity by simultaneously analyzing individual, social, and environmental factors. The model evaluated barriers and enablers of physical activity, considering correlates such as neighbourhood connectivity, safety, and living with two parents. The authors underscored the complexity of interpreting these factors, noting, for example, that higher income may reduce sedentary time overall but could also lead to increased sedentary behaviours, like watching television in bedrooms. While this study provided valuable insights into the socioecological predictors of physical activity, it did not explicitly address how systemic inequities might perpetuate differences in these behaviours.

The quality of data used in ML models remains a critical concern in mitigating bias, as models are trained on and rely heavily on dataset integrity. However, none of the studies appraised the quality of their data or explicitly discussed dataset limitations, such as the underrepresentation of specific groups. This gap may reflect the relative infancy of ML applications in population and public health. Correspondence with investigators and an analysis of bias considerations in these studies revealed that the inclusion of sociodemographic variables primarily aimed to enhance overall model performance through a “big data” approach rather than explicitly addressing algorithmic bias. Consequently, little to no discussion was provided on how algorithmic bias might influence model results or health equity outcomes (Júdice et al., 2021).

Data quality is critical in mitigating bias, as ML models inherently depend on the datasets used for training and development. However, none of the included studies thoroughly appraised dataset quality or discussed potential limitations, such as the underrepresentation of certain demographic groups. This lack of focus on data quality may reflect the relative infancy of ML applications in population and public health, where considerations of equity and inclusivity are still emerging as priorities. Addressing these gaps requires a deliberate focus on data quality and representativeness to ensure that ML applications in public health can achieve equitable and reliable outcomes.

#### Low- and middle-income country transferability

Nearly all the included studies originated from higher-income countries, with no substantive discussion of the potential for positive transferability of findings across high-income and low- and middle-income countries. This lack of consideration for LMIC applicability reflects a broader issue distinct from the typical analysis of bias: the need to account for cultural nuances and regional contexts in model development.

For example, Oku et al. (2019) leveraged data from the Brazilian National Student Health Survey (*n* = 102,301) to explore confounders in adolescent health, including unhealthy eating behaviours. The study included a culturally specific variable—“brushing teeth more than four times a day”—as a proxy for high self-care standards, reflecting a uniquely Latin American perspective on oral health. This variable emerged as the second-ranked predictor of adolescent health and was associated with broader health-related behaviours, such as eating habits. These findings underscore the importance of integrating culturally relevant variables into ML models to enhance their accuracy and applicability in diverse settings.

Furthermore, the study highlighted the complexity of LMIC contexts by analyzing regional variations within Brazil, dividing the dataset into five geopolitical regions. These regions exhibited distinct sociodemographic characteristics and were influenced by factors such as climate and natural resources. This approach emphasized the heterogeneity within LMICs and the challenges it poses for developing transferable ML models. Addressing these challenges requires careful consideration of cultural and regional factors during model design to ensure their relevance and effectiveness across diverse populations (Oku et al., 2019).

Language variations presented significant challenges for model training and development, particularly in terms of LMIC transferability. Bae et al. (2021) explored these challenges in their study on the classification of smoking status using bilingual electronic health records (EHRs). The authors highlighted that natural language processing (NLP) models designed for English benefit from the use of white spaces to define word boundaries, facilitating word tokenization and data extraction. However, in languages like Korean, the lack of clear word delimiting rules complicates tokenization, necessitating the development of entirely different NLP methodologies.

To address this, Bae et al. proposed an extraction algorithm specifically tailored for non-English-speaking countries. Their algorithm successfully classified smoking status from Korean EHRs, demonstrating that context-specific adaptations are critical for improving ML model transferability to linguistically diverse regions. This study underscores the importance of developing NLP approaches that account for linguistic and structural differences to enhance the global applicability of ML technologies in public health.

## Discussion

This scoping review examined the application of ML in identifying risk factors for NCDs. It explored whether and how biases were addressed during model development and implementation. Among the 20 included studies, we observed a predominant focus on high-income countries, limited discussion of algorithmic bias, and insufficient attention to the applicability of ML models in LMICs. By addressing multiple NCD risk factors—such as smoking, alcohol use, physical inactivity, psychological stress, and unhealthy eating—this review provides a broader synthesis than previous reviews, offering a comprehensive perspective on ML applications in public health.

Several prior reviews have explored ML applications in related domains, offering valuable context and alignment with this study. For example, Mittermaier et al. (2023a, 2023b) discussed biases in AI-based models for medical applications, emphasizing challenges such as data underrepresentation, algorithmic bias, and the need for nuanced mitigation strategies (Mittermaier et al., 2023b). Similarly, our findings reinforce these concerns, as many studies in our review superficially addressed bias, focusing primarily on traditional issues like recall and misclassification biases, while failing to discuss elements such as imbalances in data collection, which may be associated with algorithmic bias. This gap in addressing algorithmic bias reflects a broader trend noted by Mittermaier et al., who argued that failing to confront these issues can exacerbate existing health inequities (Mittermaier et al., 2023a).

However, our review diverges from  Mittermaier et al. (2023a) by focusing on public health and population-level applications rather than clinical or patient-centered contexts. For example, Mittermaier et al. (2023a) emphasized bias mitigation in diagnostic and treatment models, whereas our review considers broader applications, such as ML’s ability to model social determinants of health. This distinction is particularly evident in studies like that of Fu et al. (2022), which employed intersectionality frameworks to identify subgroups at heightened risk of vaping due to overlapping sociodemographic factors, such as perceived discrimination and income disparities. Unlike Mittermaier et al. (2023a, 2023b), we explore how these population-level insights can guide targeted interventions and inform public health policy.

Furthermore, while Mittermaier et al. (2023a) highlighted the importance of tailoring ML models to the populations they serve, their review did not emphasize the role of cultural relevance in model development. Our review extends this discussion by incorporating studies like that of Oku et al. (2019), which used culturally specific variables—such as tooth brushing frequency as a proxy for self-care—to enhance the applicability of ML models in Brazilian adolescent populations. By examining such culturally informed approaches, our review highlights the potential for ML to address public health challenges in LMICs, a focus that was underexplored in Mittermaier et al.’s work.

Overall, while both reviews underscore the critical need to address bias in ML models, our review broadens the conversation by emphasizing population-level applications, the inclusion of culturally specific variables, and the importance of equitable model development across diverse public health contexts. This broader scope complements the clinical focus of Mittermaier et al., collectively contributing to a more comprehensive understanding of how ML can be leveraged to address global health disparities.

### Strengths and limitations

A key strength of this review is its comprehensive scope, encompassing multiple NCD risk factors and exploring ML applications across varied public health domains. Unlike previous reviews that focus on specific risk factors or domains—such as those of Fu et al. (2023) on tobacco or Myrna Hurtado et al. (2022) on alcohol use—our review bridges multiple health behaviours, offering a more integrated perspective (Fu et al., 2023; Myrna Hurtado et al., 2022). Additionally, by including studies like that of Oku et al. (2019), which tailored ML applications to cultural contexts, this review underscores the potential for ML to adapt to diverse health systems and populations (Oku et al., 2019).

However, limitations exist. The overrepresentation of high-income countries (90% of included studies) limits the generalizability of findings to LMICs. Discussions of bias in the included studies were often limited to traditional biases, such as misclassification and recall bias, with little attention given to algorithmic bias. Furthermore, the exclusion of gray literature and non-English studies may have constrained the diversity of the included research. A final limitation of this review is the exclusion of studies targeting high-risk groups, specialized medical settings, and subsets of the population defined by sociodemographic characteristics other than age. While this approach was intended to enhance the generalizability of findings to the broader population, it may have overlooked critical insights about interventions in these specific subgroups. For instance, high-risk populations such as smokers or hospital patients, as well as individuals defined by characteristics like ethnicity or sex, may experience unique health needs or intervention outcomes that differ significantly from the general population (Afrifa‐Yamoah et al., 2024). Future research should address these gaps by exploring intervention effectiveness in these specialized populations to provide a more comprehensive understanding of public health strategies.

### Directions for future research

Future research should focus on addressing algorithmic bias by integrating frameworks like intersectionality, as demonstrated by Fu et al. (2022), to identify and mitigate inequities. Expanding datasets to include underrepresented populations, particularly in LMICs, is critical to enhancing the equity and applicability of models within unique cultural and social contexts. Kupek and Liberali (2024) emphasized the value of culturally specific health indicators, a principle that should guide future ML applications in global health (Kupek & Liberali, 2024).

Collaboration between high-income country and LMIC researchers is essential to ensure locally relevant model development. Tailored natural language processing methods, such as Bae et al.’s bilingual EHR algorithm, could further enhance ML’s adaptability across linguistic and cultural contexts. Additionally, longitudinal studies assessing the long-term effectiveness of ML-driven interventions on NCD outcomes would provide crucial insights into their real-world impact.

## Conclusion

This scoping review underscores ML’s transformative potential in advancing public health by predicting risk factors, tailoring interventions, and informing policies for NCD prevention. For example, Júdice et al.’s socioecological approach to physical inactivity demonstrates how ML can integrate individual, social, and environmental factors to guide interventions. Similarly, Amialchuk et al.’s analysis of adolescent alcohol use highlights ML’s role in shaping prevention strategies.

To maximize its impact, ML must address issues of bias, data quality, and cultural relevance, ensuring that models are equitable. Policymakers and public health practitioners can leverage these findings to advocate for ethical and inclusive ML practices, ultimately fostering advancements in global health equity and reducing disparities.

## Supplementary Information

Below is the link to the electronic supplementary material.

Supplementary file1 (DOCX 38 KB)

## Data Availability

The data from which the findings are generated are available upon reasonable request to the corresponding author.
