# Supplementary material for: Machine learning used to study risk factors for chronic diseases: A scoping review
Source: Can J Public Health. 2025 Jun 11;117(1):125–39. doi: 10.17269/s41997-025-01059-9 (PMC12992777; doi:10.17269/s41997-025-01059-9)
Supplement: Supplementary file 1 — Supplementary file1 (DOCX 38 KB) [file 41997_2025_1059_MOESM1_ESM.docx]

**SUPPLEMENTARY FILE 1: Complete Search Strategy**

**Database: Ovid MEDLINE: Epub Ahead of Print, In-Process & Other Non-Indexed Citations, Ovid MEDLINE® Daily and Ovid MEDLINE® <1946-Present>**

**Search Strategy:**

--------------------------------------------------------------------------------

1 artificial intelligence/ or machine learning/ or deep learning/ or supervised machine learning/ or support vector machine/ or unsupervised machine learning/ (64073)

2 computer heuristics/ or natural language processing/ or neural networks, computer/ (40225)

3 artificial intelligence.tw,kf. (18725)

4 Machine learning.tw,kf. (54771)

5 neural network*.tw,kf. (69772)

6 deep learning.tw,kf. (25183)

7 supervised learning.tw,kf. (3683)

8 unsupervised learning.tw,kf. (1877)

9 deep architecture*.tw,kf. (236)

10 computational intelligence.tw,kf. (366)

11 computer reasoning.tw,kf. (7)

12 machine intelligence.tw,kf. (198)

13 support vector machine*.tw,kf. (19832)

14 support vector network*.tw,kf. (6)

15 natural language processing.tw,kf. (5141)

16 data driven algorithm*.tw,kf. (137)

17 perceptron.tw,kf. (3021)

18 random forest*.tw,kf. (13710)

19 (ensemble learning or reinforcement learning).tw,kf. (5565)

20 AI.ti. (3112)

21 1 or 2 or 3 or 4 or 5 or 6 or 7 or 8 or 9 or 10 or 11 or 12 or 13 or 14 or 15 or 16 or 17 or 18 or 19 or 20 (189725)

22 exp Smoking/ (155663)

23 Smokers/ (3426)

24 exp Smoking Devices/ (14149)

25 Tobacco Smoke Pollution/ (14002)

26 "Tobacco Use Disorder"/ (11860)

27 exp "Tobacco Use"/ (7510)

28 smoking cessation/ or smoking reduction/ or "tobacco use cessation"/ (32113)

29 exp "Tobacco Use Cessation Devices"/ (2223)

30 Smoking Prevention/ (18420)

31 (tobacco or smoking or smoker* or vaping or vape or cigarette* or ecigarette*).tw,kf. (353364)

32 22 or 23 or 24 or 25 or 26 or 27 or 28 or 29 or 30 or 31 [smoking] (393014)

33 exp Alcohol-Related Disorders/ (117468)

34 exp Alcohol Drinking/ (74163)

35 exp Alcoholic Beverages/ (21935)

36 Alcoholics/ (865)

37 exp Alcoholics Anonymous/ (1236)

38 (alcohol* or underage drink* or binge drink* or problem drink* or at risk drink* or high risk drink* or unhealthy drinking or drinking problem* or heavy drink* or chronic drink* or daily drink* or harmful drink* or hazardous drink* or long term drink* or drinking behavior* or drinking behaviour* or heavy episodic drinking or excessive drink* or drinkers or beer drinking or wine drinking).tw,kf. (374556)

39 (drinking not drinking water).m_titl. (17625)

40 33 or 34 or 35 or 36 or 37 or 38 or 39 [problematic alcohol use] (426332)

41 Sedentary Behavior/ (11991)

42 screen time/ (798)

43 (sedentary behavior* or sedentary behaviour* or physical* inactivit* or lack of physical activity or sedentary lifestyle* or inactive lifestyle* or sedentary time* or stationary time or lack of exercise or stationary behavior* or stationary behaviour* or sitting time or screen time or sedentary work*).tw,kf. (28375)

44 sedentary.m_titl. (6605)

45 41 or 42 or 43 or 44 [physical inactivity] (34111)

46 21 and 45 (143)

47 Diet, Western/ (1228)

48 Fast Foods/ (2653)

49 exp "Feeding and Eating Disorders"/ (33546)

50 diet/ (173689)

51 Energy Intake/ (43216)

52 exp Nutrition Disorders/ (385195)

53 Sodium Chloride, Dietary/ (7619)

54 exp Dietary Sugars/ (5543)

55 dietary fats/ or cholesterol, dietary/ (55649)

56 Trans Fatty Acids/ (1199)

57 (unhealthy diet* or unhealthy eating or eating habits or fast food* or unhealthy food* or poor nutrition or poor diet* or eating disorder* or disordered eating or anorexi* or binge eating or bulim* or food addiction* or high calorie diet* or overeat* or undereating or western diet* or food intake or energy intake or food consumption or soft drink or soft drinks or junk food* or diet quality or dietary salt or salt consumption or salt intake or transfat* or trans fat* or saturated fat* or sugar consumption or sugar intake or high sugar or high fat diet* or high salt diet or sodium intake or obesogenic diet* or obesogenic food* or processed food* or fastfood* or sugary drink* or soda or sodas or portion size* or excess* sugar* or excess salt or excess* fat*).tw,kf. (235856)

58 47 or 48 or 49 or 50 or 51 or 52 or 53 or 54 or 55 or 56 or 57 [Unhealthy eating] (765511)

59 exp Stress, Psychological/ (144068)

60 (Psychologic* stress* or chronic* stress* or burnout or life stress* or high* stress* or occupational stress* or stressful or mental stress* or work related stress* or minority stress* or social* stress* or long term stress* or financial* stress* or prolonged stress* or unhealthy stress*).tw,kf. (95044)

61 (stress or stressed or stressors or stressful).ti. (270471)

62 59 or 60 or 61 [psychological stress] (405196)

63 21 and (or/22-62) (3967)

64 limit 63 to (comment or editorial or letter) (29)

65 63 not 64 (3938)

66 limit 65 to yr="2000 -Current" (3818)

67 remove duplicates from 66 (3813)

**Embase Classic+Embase <1947 to 2021 December 22>**

1 exp machine learning/ 283502

2 exp artificial intelligence/ 55169

3 natural language processing/ 7091

4 (artificial intelligence or Machine learning or neural network* or deep learning or supervised learning or unsupervised learning or deep architecture* or computational intelligence or computer reasoning or machine intelligence or support vector machine* or support vector network* or natural language processing or data driven algorithm* or perceptron or random forest* or ensemble learning or reinforcement learning).tw,kf. 191823

5 AI.m_titl. 4117

6 1 or 2 or 3 or 4 or 5 356924

7 exp "smoking and smoking related phenomena"/ 483199

8 exp smoking device/ 12206

9 smoking prevention/ 914

10 tobacco dependence/ 22574

11 (tobacco or smoking or smoker* or vaping or vape or cigarette* or ecigarette*).tw,kf. 514449

12 alcoholism/ 139386

13 exp alcohol abuse/ 43868

14 drinking behavior/ or alcohol consumption.mp. [mp=title, abstract, heading word, drug trade name, original title, device manufacturer, drug manufacturer, device trade name, keyword heading word, floating subheading word, candidate term word] 195643

15 exp alcoholic beverage/ 35239

16 alcoholics anonymous/ 1841

17 (alcohol* or underage drink* or binge drink* or problem drink* or at risk drink* or high risk drink* or unhealthy drinking or drinking problem* or heavy drink* or chronic drink* or daily drink* or harmful drink* or hazardous drink* or long term drink* or drinking behavior* or drinking behaviour* or heavy episodic drinking or excessive drink* or drinkers or beer drinking or wine drinking).tw,kf. 535490

18 (drinking not drinking water).m_titl. 22514

19 sedentary lifestyle/ 16829

20 sedentary time/ 1743

21 (sedentary behavior* or sedentary behaviour* or physical* inactivit* or lack of physical activity or sedentary lifestyle* or inactive lifestyle* or sedentary time* or stationary time or lack of exercise or stationary behavior* or stationary behaviour* or sitting time or screen time or sedentary work* or time spent sitting).tw,kf. 37360

22 sedentary.m_titl. 7886

23 unhealthy diet/ 1216

24 fast food/ 9076

25 junk food/ 400

26 convenience food/ 421

27 processed food/ or ultra-processed food/ 1099

28 exp eating disorder/ 58888

29 exp dietary intake/ 613452

30 (unhealthy diet* or unhealthy eating or eating habits or fast food* or unhealthy food* or poor nutrition or poor diet* or eating disorder* or disordered eating or anorexi* or binge eating or bulim* or food addiction* or high calorie diet* or overeat* or undereating or western diet* or food intake or energy intake or food consumption or soft drink or soft drinks or junk food* or diet quality or dietary salt or salt consumption or salt intake or transfat* or trans fat* or saturated fat* or sugar consumption or sugar intake or high sugar or high fat diet* or high salt diet or sodium intake or obesogenic diet* or obesogenic food* or processed food* or fastfood* or sugary drink* or soda or sodas or portion size* or excess* sugar* or excess salt or excess* fat*).tw,kf. 326210

31 exp chronic stress/ 12222

32 exp mental stress/ 177479

33 (Psychologic* stress* or chronic* stress* or burnout or life stress* or high* stress* or occupational stress* or stressful or mental stress* or work related stress* or minority stress* or social* stress* or long term stress* or financial* stress* or prolonged stress* or unhealthy stress*).tw,kf. 123839

34 (stress or stressed or stressors or stressful).ti. 327472

35 or/7-34 2329672

36 6 and 35 9733

37 limit 36 to (chapter or conference abstract or conference paper or "conference review" or editorial or letter) 2375

38 36 not 37 7358

39 38 not ((exp animal/ or animal experiment/ or nonhuman/) not (exp human/ or human experiment/)) 5961

40 limit 39 to embase 4758

41 limit 40 to yr="2000 -Current" 4665

42 remove duplicates from 41 4649

**EBM Reviews - Cochrane Central Register of Controlled Trials <November 2021>**

**EBM Reviews - Cochrane Database of Systematic Reviews <2005 to December 22, 2021>**

1 artificial intelligence/ or machine learning/ or deep learning/ or supervised machine learning/ or support vector machine/ or unsupervised machine learning/ 378

2 computer heuristics/ or natural language processing/ or neural networks, computer/ 136

3 (artificial intelligence or Machine learning or neural network* or deep learning or supervised learning or unsupervised learning or deep architecture* or computational intelligence or computer reasoning or machine intelligence or support vector machine* or support vector network* or natural language processing or data driven algorithm* or perceptron or random forest* or ensemble learning or reinforcement learning).tw. 4611

4 AI.m_titl. 312

5 1 or 2 or 3 or 4 5013

6 exp Smoking/ or Smokers/ or exp Smoking Devices/ or Tobacco Smoke Pollution/ or "Tobacco Use Disorder"/ or exp "Tobacco Use"/ or smoking cessation/ or smoking reduction/ or "tobacco use cessation"/ or exp "Tobacco Use Cessation Devices"/ or Smoking Prevention/ 9995

7 (tobacco or smoking or smoker* or vaping or vape or cigarette* or ecigarette*).tw. 41495

8 exp Alcohol-Related Disorders/ or exp Alcohol Drinking/ or exp Alcoholic Beverages/ or Alcoholics/ or exp Alcoholics Anonymous/ 8280

9 (alcohol* or underage drink* or binge drink* or problem drink* or at risk drink* or high risk drink* or unhealthy drinking or drinking problem* or heavy drink* or chronic drink* or daily drink* or harmful drink* or hazardous drink* or long term drink* or drinking behavior* or drinking behaviour* or heavy episodic drinking or excessive drink* or drinkers or beer drinking or wine drinking).tw. 33364

10 (drinking not drinking water).m_titl. 1943

11 Sedentary Behavior/ or screen time/ 1310

12 (sedentary behavior* or sedentary behaviour* or physical* inactivit* or lack of physical activity or sedentary lifestyle* or inactive lifestyle* or sedentary time* or stationary time or lack of exercise or stationary behavior* or stationary behaviour* or sitting time or screen time or sedentary work*).tw. 5485

13 sedentary.m_titl. 1564

14 Diet, Western/ or Fast Foods/ or exp "Feeding and Eating Disorders"/ or diet/ or Energy Intake/ or exp Nutrition Disorders/ or Sodium Chloride, Dietary/ or exp Dietary Sugars/ or dietary fats/ or cholesterol, dietary/ or Trans Fatty Acids/ 31956

15 (unhealthy diet* or unhealthy eating or eating habits or fast food* or unhealthy food* or poor nutrition or poor diet* or eating disorder* or disordered eating or anorexi* or binge eating or bulim* or food addiction* or high calorie diet* or overeat* or undereating or western diet* or food intake or energy intake or food consumption or soft drink or soft drinks or junk food* or diet quality or dietary salt or salt consumption or salt intake or transfat* or trans fat* or saturated fat* or sugar consumption or sugar intake or high sugar or high fat diet* or high salt diet or sodium intake or obesogenic diet* or obesogenic food* or processed food* or fastfood* or sugary drink* or soda or sodas or portion size* or excess* sugar* or excess salt or excess* fat*).tw. 27712

16 exp Stress, Psychological/ 6443

17 (Psychologic* stress* or chronic* stress* or burnout or life stress* or high* stress* or occupational stress* or stressful or mental stress* or work related stress* or minority stress* or social* stress* or long term stress* or financial* stress* or prolonged stress* or unhealthy stress*).tw. 8509

18 (stress or stressed or stressors or stressful).ti. 19858

19 or/6-18 151372

20 5 and 19 319

21 limit 20 to yr="2000 -Current" 275

22 remove duplicates from 21 271

**Search History**

Interface - EBSCOhost Research Databases
Search Screen - Advanced Search
**Database - CINAHL Complete**

| \| **#** \| **Query** \| **Limiters/Expanders** \| **Results** \| \| --- \| --- \| --- \| --- \| \| S41 \| S39 NOT S40 \| Limiters - Published Date: 20000101-20221231 Expanders - Apply equivalent subjects Search modes - Boolean/Phrase \| 957 \| \| S40 \| TI ( (animal or animals or canine* or dog or dogs or feline or hamster* or lamb or lambs or mice or monkey or monkeys or mouse or murine or pig or pigs or piglet* or porcine or primate* or rabbit* or rats or rat or rodent* or sheep* ) NOT (human* or patient*)) \| Expanders - Apply equivalent subjects Search modes - Boolean/Phrase \| 109,356 \| \| S39 \| S37 NOT S38 \| Expanders - Apply equivalent subjects Search modes - Boolean/Phrase \| 980 \| \| S38 \| S37 \| Limiters - Publication Type: Book, Book Chapter, Book Review, Commentary, Doctoral Dissertation, Editorial, Letter, Masters Thesis, Proceedings, Response Expanders - Apply equivalent subjects Search modes - Boolean/Phrase \| 66 \| \| S37 \| S4 AND S36 \| Expanders - Apply equivalent subjects Search modes - Boolean/Phrase \| 1,046 \| \| S36 \| S5 OR S6 OR S7 OR S8 OR S9 OR S10 OR S11 OR S12 OR S13 OR S14 OR S15 OR S16 OR S17 OR S18 OR S19 OR S20 OR S21 OR S22 OR S23 OR S24 OR S25 OR S26 OR S27 OR S28 OR S29 OR S30 OR S31 OR S32 OR S33 OR S34 OR S35 \| Expanders - Apply equivalent subjects Search modes - Boolean/Phrase \| 644,101 \| \| S35 \| TI (stress or stressed or stressors or stressful). \| Expanders - Apply equivalent subjects Search modes - Boolean/Phrase \| 61,660 \| \| S34 \| (Psychologic* stress* or chronic* stress* or burnout or life stress* or high* stress* or occupational stress* or stressful or mental stress* or work related stress* or minority stress* or social* stress* or long term stress* or financial* stress* or prolonged stress* or unhealthy stress*) \| Expanders - Apply equivalent subjects Search modes - Boolean/Phrase \| 72,105 \| \| S33 \| (MH "Stress+") \| Expanders - Apply equivalent subjects Search modes - Boolean/Phrase \| 107,967 \| \| S32 \| (unhealthy diet* or unhealthy eating or eating habits or fast food* or unhealthy food* or poor nutrition or poor diet* or eating disorder* or disordered eating or anorexi* or binge eating or bulim* or food addiction* or high calorie diet* or overeat* or undereating or western diet* or food intake or energy intake or food consumption or soft drink or soft drinks or junk food* or diet quality or dietary salt or salt consumption or salt intake or transfat* or trans fat* or saturated fat* or sugar consumption or sugar intake or high sugar or high fat diet* or high salt diet or sodium intake or obesogenic diet* or obesogenic food* or processed food* or fastfood* or sugary drink* or soda or sodas or portion size* or excess* sugar* or excess salt or excess* fat*) \| Expanders - Apply equivalent subjects Search modes - Boolean/Phrase \| 108,580 \| \| S31 \| (MH "Trans Fatty Acids") \| Expanders - Apply equivalent subjects Search modes - Boolean/Phrase \| 1,300 \| \| S30 \| (MH "Cholesterol, Dietary") OR (MH "Dietary Fats") \| Expanders - Apply equivalent subjects Search modes - Boolean/Phrase \| 15,274 \| \| S29 \| (MH "Dietary Sucrose") OR (MH "High Fructose Corn Syrup") \| Expanders - Apply equivalent subjects Search modes - Boolean/Phrase \| 4,925 \| \| S28 \| (MH "Sodium Chloride, Dietary") \| Expanders - Apply equivalent subjects Search modes - Boolean/Phrase \| 3,852 \| \| S27 \| (MH "Nutrition Disorders+") \| Expanders - Apply equivalent subjects Search modes - Boolean/Phrase \| 145,206 \| \| S26 \| (MH "Energy Intake") \| Expanders - Apply equivalent subjects Search modes - Boolean/Phrase \| 19,858 \| \| S25 \| (MH "Diet") \| Expanders - Apply equivalent subjects Search modes - Boolean/Phrase \| 61,156 \| \| S24 \| (MH "Eating Disorders+") \| Expanders - Apply equivalent subjects Search modes - Boolean/Phrase \| 19,711 \| \| S23 \| (MH "Food Addiction") \| Expanders - Apply equivalent subjects Search modes - Boolean/Phrase \| 315 \| \| S22 \| (MH "Fast Foods") \| Expanders - Apply equivalent subjects Search modes - Boolean/Phrase \| 1,398 \| \| S21 \| (MH "Diet, Western") \| Expanders - Apply equivalent subjects Search modes - Boolean/Phrase \| 566 \| \| S20 \| TI sedentary \| Expanders - Apply equivalent subjects Search modes - Boolean/Phrase \| 3,868 \| \| S19 \| (sedentary behavior* or sedentary behaviour* or physical* inactivit* or lack of physical activity or sedentary lifestyle* or inactive lifestyle* or sedentary time* or stationary time or lack of exercise or stationary behavior* or stationary behaviour* or sitting time or screen time or sedentary work*) \| Expanders - Apply equivalent subjects Search modes - Boolean/Phrase \| 15,544 \| \| S18 \| (MH "Life Style, Sedentary+") \| Expanders - Apply equivalent subjects Search modes - Boolean/Phrase \| 9,476 \| \| S17 \| TI drinking NOT drinking water \| Expanders - Apply equivalent subjects Search modes - Boolean/Phrase \| 8,624 \| \| S16 \| (alcohol* or underage drink* or binge drink* or problem drink* or at risk drink* or high risk drink* or unhealthy drinking or drinking problem* or heavy drink* or chronic drink* or daily drink* or harmful drink* or hazardous drink* or long term drink* or drinking behavior* or drinking behaviour* or heavy episodic drinking or excessive drink* or drinkers or beer drinking or wine drinking) \| Expanders - Apply equivalent subjects Search modes - Boolean/Phrase \| 118,819 \| \| S15 \| (MH "Alcoholic Beverages+") \| Expanders - Apply equivalent subjects Search modes - Boolean/Phrase \| 5,716 \| \| S14 \| (MH "Alcohol Rehabilitation Programs+") \| Expanders - Apply equivalent subjects Search modes - Boolean/Phrase \| 2,669 \| \| S13 \| (MH "Alcohol Rehabilitation Programs+") \| Expanders - Apply equivalent subjects Search modes - Boolean/Phrase \| 2,669 \| \| S12 \| (MH "Alcohol Rehabilitation Programs+") \| Expanders - Apply equivalent subjects Search modes - Boolean/Phrase \| 2,669 \| \| S11 \| (MH "Drinking Behavior+") \| Expanders - Apply equivalent subjects Search modes - Boolean/Phrase \| 35,491 \| \| S10 \| (MH "Alcohol-Related Disorders+") \| Expanders - Apply equivalent subjects Search modes - Boolean/Phrase \| 39,557 \| \| S9 \| (tobacco or smoking or smoker* or vaping or vape or cigarette* or ecigarette*) \| Expanders - Apply equivalent subjects Search modes - Boolean/Phrase \| 138,595 \| \| S8 \| (MH "Smoking Cessation Programs") \| Expanders - Apply equivalent subjects Search modes - Boolean/Phrase \| 2,544 \| \| S7 \| (MH "Tobacco Products+") OR (MH "Tobacco Use Cessation Products+") \| Expanders - Apply equivalent subjects Search modes - Boolean/Phrase \| 8,446 \| \| S6 \| (MM "Tobacco") \| Expanders - Apply equivalent subjects Search modes - Boolean/Phrase \| 5,274 \| \| S5 \| (MH "Smoking+") \| Expanders - Apply equivalent subjects Search modes - Boolean/Phrase \| 76,112 \| \| S4 \| S1 OR S2 OR S3 \| Expanders - Apply equivalent subjects Search modes - Boolean/Phrase \| 28,162 \| \| S3 \| TI AI \| Expanders - Apply equivalent subjects Search modes - Boolean/Phrase \| 1,568 \| \| S2 \| (artificial intelligence or Machine learning or neural network* or deep learning or supervised learning or unsupervised learning or deep architecture* or computational intelligence or computer reasoning or machine intelligence or support vector machine* or support vector network* or natural language processing or data driven algorithm* or perceptron or random forest* or ensemble learning or reinforcement learning) \| Expanders - Apply equivalent subjects Search modes - Boolean/Phrase \| 27,012 \| \| S1 \| (MH "Artificial Intelligence") OR (MH "Expert Systems") OR (MH "Knowbots") OR (MH "Machine Learning+") OR (MH "Natural Language Processing") OR (MH "Neural Networks (Computer)") \| Expanders - Apply equivalent subjects Search modes - Boolean/Phrase \| 13,338 \| |
| --- | --- | --- | --- | --- | --- | --- | --- | --- | --- | --- | --- | --- | --- | --- | --- | --- | --- | --- | --- | --- | --- | --- | --- | --- | --- | --- | --- | --- | --- | --- | --- | --- | --- | --- | --- | --- | --- | --- | --- | --- | --- | --- | --- | --- | --- | --- | --- | --- | --- | --- | --- | --- | --- | --- | --- | --- | --- | --- | --- | --- | --- | --- | --- | --- | --- | --- | --- | --- | --- | --- | --- | --- | --- | --- | --- | --- | --- | --- | --- | --- | --- | --- | --- | --- | --- | --- | --- | --- | --- | --- | --- | --- | --- | --- | --- | --- | --- | --- | --- | --- | --- | --- | --- | --- | --- | --- | --- | --- | --- | --- | --- | --- | --- | --- | --- | --- | --- | --- | --- | --- | --- | --- | --- | --- | --- | --- | --- | --- | --- | --- | --- | --- | --- | --- | --- | --- | --- | --- | --- | --- | --- | --- | --- | --- | --- | --- | --- | --- | --- | --- | --- | --- | --- | --- | --- | --- | --- | --- | --- | --- | --- | --- | --- | --- | --- | --- | --- | --- |

**Scopus 837 Results**

( ( TITLE-ABS ( ( "artificial intelligence" OR "Machine learning" OR "neural network* " OR "deep learning " OR "supervised learning " OR "unsupervised learning " OR "deep architecture* " OR "computational intelligence " OR "computer reasoning " OR "machine intelligence " OR "support vect machine*" OR "support vector network* " OR "natural language processing " OR "data driven algorithm* " OR perceptron OR "random forest* " OR "ensemble learning " OR "reinforcement learning" ) ) ) OR ( TITLE ( ai ) ) ) AND ( ( TITLE-ABS ( ( tobacco OR smoking OR smoker* OR vaping OR vape OR cigarette* OR ecigarette* ) ) ) OR ( TITLE-ABS ( ( alcoholism or alcoholic* OR "underage drink* " OR "binge drink* " OR "problem drink* " OR "at risk drink* " OR "high risk drink* " OR "unhealthy drinking " OR "drinking problem* " OR "heavy drink* " OR "chronic drink* " OR "daily drink* " OR "harmful drink* " OR "hazardous drink* " OR "long term drink* " OR "drinking behavior* " OR "drinking behaviour* " OR "heavy episodic drinking " OR "excessive drink* " OR drinkers OR "beer drinking " OR "wine drinking" ) ) ) OR ( ( TITLE ( drinking ) AND NOT TITLE ( "drinking water" ) ) ) OR ( ( TITLE-ABS ( ( "sedentary behavior* " OR "sedentary behaviour* " OR "physical* inactivit* " OR "lack of physical activity " OR "sedentary lifestyle* " OR "inactive lifestyle* " OR "sedentary time* " OR "stationary time " OR "lack of exercise " OR "stationary behavior* " OR "stationary behaviour* " OR "sitting time " OR "screen time " OR "sedentary work*" ) ) OR TITLE ( sedentary ) ) ) OR ( TITLE-ABS ( ( "unhealthy diet* " OR "unhealthy eating " OR "eating habits " OR "fast food* " OR "unhealthy food* " OR "poor nutrition " OR "poor diet* " OR "eating disorder* " OR "disordered eating " OR anorexi* OR "binge eating " OR bulim* OR "food addiction* " OR "high calorie diet* " OR overeat* OR undereating OR "western diet* " OR "food intake " OR "food consumption " OR "soft drink " OR "soft drinks " OR "junk food* " OR "diet quality " OR "dietary salt " OR "salt consumption " OR "sugar consumption " OR "sugar intake " OR "high sugar diet " OR "high fat diet* " OR "high salt diet " OR "sodium intake " OR "obesogenic diet* " OR "obesogenic food* " OR "processed food* " OR fastfood* OR "sugary drink* " OR soda OR sodas OR "portion size* " OR "excess* sugar* " OR "excess salt " OR "excess* fat*" ) ) ) OR ( ( TITLE-ABS ( ( "Psychologic* stress* " OR "chronic* stress* " OR burnout OR "life stress* " OR "high* stress* " OR "occupational stress* " OR "mental stress* " OR "work related stress* " OR "minority stress* " OR "social* stress* " OR "long term stress* " OR "financial* stress* " OR "prolonged stress* " OR "unhealthy stress*" ) ) OR TITLE ( (stressful ) ) ) ) ) AND NOT ( TITLE ( animal* OR nonhuman* OR veterinar* OR avian* OR baboon* OR bird* OR bovine OR canine OR cat OR cats OR cattle* OR chick* OR chimp* OR cow OR cows OR dog OR dogs OR duck OR feline OR fish* OR geese OR goose OR macaque* OR marmoset* OR mice OR mouse OR murine OR ovine OR pig OR pigs OR piglet* OR porcine OR primate* OR rabbit OR rat OR rats OR rodent* OR sheep OR swine OR trout* OR zebrafish* ) AND NOT ( human* OR patient* OR women OR woman OR men OR man ) ) AND NOT INDEX ( medline ) AND ( LIMIT-TO ( PUBYEAR , 2022 ) OR LIMIT-TO ( PUBYEAR , 2021 ) OR LIMIT-TO ( PUBYEAR , 2020 ) OR LIMIT-TO ( PUBYEAR , 2019 ) OR LIMIT-TO ( PUBYEAR , 2018 ) OR LIMIT-TO ( PUBYEAR , 2017 ) OR LIMIT-TO ( PUBYEAR , 2016 ) OR LIMIT-TO ( PUBYEAR , 2015 ) OR LIMIT-TO ( PUBYEAR , 2014 ) OR LIMIT-TO ( PUBYEAR , 2013 ) OR LIMIT-TO ( PUBYEAR , 2012 ) OR LIMIT-TO ( PUBYEAR , 2011 ) OR LIMIT-TO ( PUBYEAR , 2010 ) OR LIMIT-TO ( PUBYEAR , 2009 ) OR LIMIT-TO ( PUBYEAR , 2008 ) OR LIMIT-TO ( PUBYEAR , 2007 ) OR LIMIT-TO ( PUBYEAR , 2006 ) OR LIMIT-TO ( PUBYEAR , 2005 ) OR LIMIT-TO ( PUBYEAR , 2004 ) OR LIMIT-TO ( PUBYEAR , 2003 ) OR LIMIT-TO ( PUBYEAR , 2002 ) OR LIMIT-TO ( PUBYEAR , 2001 ) OR LIMIT-TO ( PUBYEAR , 2000 ) ) AND ( EXCLUDE ( DOCTYPE , "cp" ) OR EXCLUDE ( DOCTYPE , "ch" ) OR EXCLUDE ( DOCTYPE , "cr" ) OR EXCLUDE ( DOCTYPE , "bk" ) OR EXCLUDE ( DOCTYPE , "no" ) OR EXCLUDE ( DOCTYPE , "ed" ) OR EXCLUDE ( DOCTYPE , "dp" ) OR EXCLUDE ( DOCTYPE , "le" ) OR EXCLUDE ( DOCTYPE , "ab" ) ) AND ( EXCLUDE ( SUBJAREA , "CHEM" ) OR EXCLUDE ( SUBJAREA , "PHYS" ) OR EXCLUDE ( SUBJAREA , "CENG" ) OR EXCLUDE ( SUBJAREA , "EART" ) OR EXCLUDE ( SUBJAREA , "ENER" ) OR EXCLUDE ( SUBJAREA , "VETE" ) OR EXCLUDE ( SUBJAREA , "DENT" ) ) AND ( EXCLUDE ( EXACTKEYWORD , "Nonhuman" ) OR EXCLUDE ( EXACTKEYWORD , "Residual Stresses" ) OR EXCLUDE ( EXACTKEYWORD , "Oxidative Stress" ) OR EXCLUDE ( EXACTKEYWORD , "Animal Experiment" ) OR EXCLUDE ( EXACTKEYWORD , "Residual Stress" ) OR EXCLUDE ( EXACTKEYWORD , "Rat" ) OR EXCLUDE ( EXACTKEYWORD , "Abiotic Stress" ) OR EXCLUDE ( EXACTKEYWORD , "Fatigue Of Materials" ) OR EXCLUDE ( EXACTKEYWORD , "Mouse" ) OR EXCLUDE ( EXACTKEYWORD , "Enzyme Activity" ) OR EXCLUDE ( EXACTKEYWORD , "Cracks" ) OR EXCLUDE ( EXACTKEYWORD , "Polyvinyl Alcohols" ) OR EXCLUDE ( EXACTKEYWORD , "Drought Stress" ) OR EXCLUDE ( EXACTKEYWORD , "Strain Rate" ) OR EXCLUDE ( EXACTKEYWORD , "Environmental Stress" ) OR EXCLUDE ( EXACTKEYWORD , "Tensile Strength" ) OR EXCLUDE ( EXACTKEYWORD , "Animal Cell" ) OR EXCLUDE ( EXACTKEYWORD , "Poly (vinyl Alcohol) (PVA)" ) OR EXCLUDE ( EXACTKEYWORD , "Drought" ) OR EXCLUDE ( EXACTKEYWORD , "Machinery" ) OR EXCLUDE ( EXACTKEYWORD , "Reactive Oxygen Metabolite" ) OR EXCLUDE ( EXACTKEYWORD , "Hydrogels" ) OR EXCLUDE ( EXACTKEYWORD , "Plants (botany)" ) OR EXCLUDE ( EXACTKEYWORD , "Aluminum Alloys" ) OR EXCLUDE ( EXACTKEYWORD , "Reactive Oxygen Species" ) OR EXCLUDE ( EXACTKEYWORD , "Salinity" ) OR EXCLUDE ( EXACTKEYWORD , "Photosynthesis" ) OR EXCLUDE ( EXACTKEYWORD , "Plastic Flow" ) OR EXCLUDE ( EXACTKEYWORD , "Shear Stress" ) OR EXCLUDE ( EXACTKEYWORD , "Temperature" ) ) AND ( EXCLUDE ( SUBJAREA , "MATE" ) OR EXCLUDE ( SUBJAREA , "ENVI" ) OR EXCLUDE ( SUBJAREA , "BUSI" ) OR EXCLUDE ( SUBJAREA , "ARTS" ) OR EXCLUDE ( SUBJAREA , "ECON" ) ) AND ( LIMIT-TO ( SRCTYPE , "j" ) )

**Web of Science**

Editions = ESCI , SCI-EXPANDED , SSCI

| # | Search History | Results |
| --- | --- | --- |
| 1 | (TI=("artificial intelligence" OR "Machine learning" OR "neural network* " OR "deep learning " OR "supervised learning " OR "unsupervised learning " OR "deep architecture* " OR "computational intelligence " OR "computer reasoning " OR "machine intelligence " OR "support vect machine*" OR "support vector network* " OR "natural language processing " OR "data driven algorithm* " OR perceptron OR "random forest* " OR "ensemble learning " OR "reinforcement learning") OR AB=("artificial intelligence" OR "Machine learning" OR "neural network* " OR "deep learning " OR "supervised learning " OR "unsupervised learning " OR "deep architecture* " OR "computational intelligence " OR "computer reasoning " OR "machine intelligence " OR "support vect machine*" OR "support vector network* " OR "natural language processing " OR "data driven algorithm* " OR perceptron OR "random forest* " OR "ensemble learning " OR "reinforcement learning") OR TI=(AI) ) NOT TI=( animal* OR nonhuman* OR veterinar* OR avian* OR baboon* OR bird* OR bovine OR canine OR cat OR cats OR cattle* OR chick* OR chimp* OR cow OR cows OR dog OR dogs OR duck OR feline OR fish* OR geese OR goose OR macaque* OR marmoset* OR mice OR mouse OR murine OR ovine OR pig OR pigs OR piglet* OR porcine OR primate* OR rabbit OR rat OR rats OR rodent* OR sheep OR swine OR trout* OR zebrafish* ) | 435,798 |
| 2 | ( tobacco OR smoking OR smoker* OR vaping OR vape OR cigarette* OR ecigarette* OR alcoholism or alcoholic* OR "underage drink* " OR "binge drink* " OR "problem drink* " OR "at risk drink* " OR "high risk drink* " OR "unhealthy drinking " OR "drinking problem* " OR "heavy drink* " OR "chronic drink* " OR "daily drink* " OR "harmful drink* " OR "hazardous drink* " OR "long term drink* " OR "drinking behavior* " OR "drinking behaviour* " OR "heavy episodic drinking " OR "excessive drink* " OR drinkers OR "beer drinking " OR "wine drinking" OR "sedentary behavior* " OR "sedentary behaviour* " OR "physical* inactivit* " OR "lack of physical activity " OR "sedentary lifestyle* " OR "inactive lifestyle* " OR "sedentary time* " OR "stationary time " OR "lack of exercise " OR "stationary behavior* " OR "stationary behaviour* " OR "sitting time " OR "screen time " OR "sedentary work*" OR "unhealthy diet* " OR "unhealthy eating " OR "eating habits " OR "fast food* " OR "unhealthy food* " OR "poor nutrition " OR "poor diet* " OR "eating disorder* " OR "disordered eating " OR anorexi* OR "binge eating " OR bulim* OR "food addiction* " OR "high calorie diet* " OR overeat* OR undereating OR "western diet* " OR "food intake " OR "food consumption " OR "soft drink " OR "soft drinks " OR "junk food* " OR "diet quality " OR "dietary salt " OR "salt consumption " OR "sugar consumption " OR "sugar intake " OR "high sugar diet " OR "high fat diet* " OR "high salt diet " OR "sodium intake " OR "obesogenic diet* " OR "obesogenic food* " OR "processed food* " OR fastfood* OR "sugary drink* " OR soda OR sodas OR "portion size* " OR "excess* sugar* " OR "excess salt " OR "excess* fat*" OR "Psychologic* stress* " OR "chronic* stress* " OR burnout OR "life stress* " OR "high* stress* " OR "occupational stress* " OR "mental stress* " OR "work related stress* " OR "minority stress* " OR "social* stress* " OR "long term stress* " OR "financial* stress* " OR "prolonged stress* " OR "unhealthy stress*") (Title) or ( tobacco OR smoking OR smoker* OR vaping OR vape OR cigarette* OR ecigarette* OR alcoholism or alcoholic* OR "underage drink* " OR "binge drink* " OR "problem drink* " OR "at risk drink* " OR "high risk drink* " OR "unhealthy drinking " OR "drinking problem* " OR "heavy drink* " OR "chronic drink* " OR "daily drink* " OR "harmful drink* " OR "hazardous drink* " OR "long term drink* " OR "drinking behavior* " OR "drinking behaviour* " OR "heavy episodic drinking " OR "excessive drink* " OR drinkers OR "beer drinking " OR "wine drinking" OR "sedentary behavior* " OR "sedentary behaviour* " OR "physical* inactivit* " OR "lack of physical activity " OR "sedentary lifestyle* " OR "inactive lifestyle* " OR "sedentary time* " OR "stationary time " OR "lack of exercise " OR "stationary behavior* " OR "stationary behaviour* " OR "sitting time " OR "screen time " OR "sedentary work*" OR "unhealthy diet* " OR "unhealthy eating " OR "eating habits " OR "fast food* " OR "unhealthy food* " OR "poor nutrition " OR "poor diet* " OR "eating disorder* " OR "disordered eating " OR anorexi* OR "binge eating " OR bulim* OR "food addiction* " OR "high calorie diet* " OR overeat* OR undereating OR "western diet* " OR "food intake " OR "food consumption " OR "soft drink " OR "soft drinks " OR "junk food* " OR "diet quality " OR "dietary salt " OR "salt consumption " OR "sugar consumption " OR "sugar intake " OR "high sugar diet " OR "high fat diet* " OR "high salt diet " OR "sodium intake " OR "obesogenic diet* " OR "obesogenic food* " OR "processed food* " OR fastfood* OR "sugary drink* " OR soda OR sodas OR "portion size* " OR "excess* sugar* " OR "excess salt " OR "excess* fat*" OR "Psychologic* stress* " OR "chronic* stress* " OR burnout OR "life stress* " OR "high* stress* " OR "occupational stress* " OR "mental stress* " OR "work related stress* " OR "minority stress* " OR "social* stress* " OR "long term stress* " OR "financial* stress* " OR "prolonged stress* " OR "unhealthy stress*") (Abstract) | 861,411 |
| 3 | drinking (Title) not "drinking water" (All Fields) | 36,889 |
| 4 | sedentary (Title) or (stress or stressed or stressors or stressful) (Title) | 540,369 |
| 5 | #4 OR #3 OR #2 | 1,369,312 |
| 6 | #4 OR #3 OR #2  Timespan: 2000-01-01 to 2022-12-12 (Publication Date) | 1,069,546 |
| 7 | #6 AND #1  Timespan: 2000-01-01 to 2022-12-12 (Publication Date) | 3,632 |
| 8 | #6 AND #1 and Proceedings Papers or Meeting Abstracts or Book Chapters or Letters or News Items or Editorial Materials (Exclude – Document Types)  Timespan: 2000-01-01 to 2022-12-12 (Publication Date) | 3,422 |
|  | #6 AND #1 and Proceedings Papers or Meeting Abstracts or Book Chapters or Letters or News Items or Editorial Materials (Exclude – Document Types) and Mineralogy or Fisheries or Crystallography or Biodiversity Conservation or Astronomy Astrophysics or Oceanography or Nuclear Science Technology or Polymer Science or Mining Mineral Processing or Zoology or Dentistry Oral Surgery Medicine or Acoustics or Marine Freshwater Biology or Physical Geography or Forestry or Veterinary Sciences or Transportation or Geochemistry Geophysics or Thermodynamics or Meteorology Atmospheric Sciences or Optics or Construction Building Technology or Water Resources or Business Economics or Environmental Sciences Ecology or Agriculture or Metallurgy Metallurgical Engineering or Energy Fuels or Geology or Mechanics or Plant Sciences (Exclude – Research Areas)  Timespan: 2000-01-01 to 2022-12-12 (Publication Date) | 2,726 |

**ACM Digital Library (Association for Computing Machinery)**

[[Title: "neural network "] OR [Title: "deep learning "] OR [Title: "supervised learning "] OR [Title: "unsupervised learning "] OR [Title: "deep architecture* "] OR [Title: "computational intelligence "] OR [Title: "computer reasoning "] OR [Title: "machine intelligence "] OR [Title: "support vect machine"] OR [Title: "support vector network "] OR [Title: "natural language processing "] OR [Title: "data driven algorithm "] OR [Title: perceptron] OR [Title: "random forest "] OR [Title: "ensemble learning "] OR [Title: "reinforcement learning"]] AND [[Title: smoking] OR [Title: drinking] OR [Title: diet] OR [Title: stress or sedentary]] AND [Publication Date: (01/01/2000 TO 31/12/2021)]

[[Abstract: "neural network "] OR [Abstract: "deep learning "] OR [Abstract: "supervised learning "] OR [Abstract: "unsupervised learning "] OR [Abstract: "deep architecture* "] OR [Abstract: "computational intelligence "] OR [Abstract: "computer reasoning "] OR [Abstract: "machine intelligence "] OR [Abstract: "support vect machine"] OR [Abstract: "support vector network "] OR [Abstract: "natural language processing "] OR [Abstract: "data driven algorithm "] OR [Abstract: perceptron] OR [Abstract: "random forest "] OR [Abstract: "ensemble learning "] OR [Abstract: "reinforcement learning"]] AND [[Abstract: smoking] OR [Abstract: drinking] OR [Abstract: diet] OR [Abstract: stress or sedentary]] AND [Publication Date: (01/01/2000 TO 31/12/2021)]

___

[[Abstract: "machine learning"] OR [Abstract: "artifical intelligence"]] AND [[Abstract: smoking] OR [Abstract: drinking] OR [Abstract: diet] OR [Abstract: stress or sedentary]] AND [Publication Date: (01/01/2000 TO 31/12/2021)]

[[Title: "machine learning"] OR [Title: "artifical intelligence"]] AND [[Title: smoking] OR [Title: drinking] OR [Title: diet] OR [Title: stress or sedentary]] AND [Publication Date: (01/01/2000 TO 31/12/2021)]

**Inspec (Engineering Village/Elsevier)**

2,784 records found in Inspec for :

(( ((($smoking OR $smoker OR $smokers OR $drinking OR $alcoholic OR $drinker OR $drinkers OR $alcoholism OR $sedentary OR {screen time} OR $diet OR $dietary OR $stress OR $burnout)) WN KY) AND (JA WN DT) AND (2000-2022 WN YR)) AND ( (((({artificial intelligence} OR {Machine learning} OR {neural network } OR {neural networks } OR {deep learning } OR {supervised learning } OR {unsupervised learning } OR {deep architecture } OR {computational intelligence } OR {computer reasoning } OR {machine intelligence } OR {support vector machine} OR {support vector network } OR {natural language processing } OR {data driven algorithm } OR $perceptron OR {random forest* } OR {ensemble learning } OR {reinforcement learning}) WN KY) OR (({learning (artificial intelligence)}) WN CV)) OR (($AI) WN TI)) AND (JA WN DT) AND (2000-2022 WN YR))) - ({stress-strain relations} OR {aluminium alloys} OR {mechanical engineering computing} OR {structural engineering computing} OR {geotechnical engineering} OR {tensile strength} OR {rocks} OR {compressive strength} OR {magnesium alloys} OR {crystal microstructure}) WN CV - ({e1710} OR {a9385} OR {e3010} OR {c7860} OR {e1525} OR {a4710} OR {c7340} OR {e2130} OR {a9240q}) WN CL
